# Endometrial hyperplasia as a risk factor of endometrial cancer

**DOI:** 10.1007/s00404-021-06380-5

**Published:** 2022-01-10

**Authors:** Lisa K. Nees, Sabine Heublein, Sahra Steinmacher, Ingolf Juhasz-Böss, Sara Brucker, Clemens B. Tempfer, Markus Wallwiener

**Affiliations:** 1 Department of Obstetrics and Gynecology, University of Heidelberg, Im Neuenheimer Feld 440, 69120 Heidelberg, Germany; 2 Department of Obstetrics and Gynecology, Universität Tübingen, Tübingen, Germany; 3 Department of Obstetrics and Gynecology, Universität Freiburg, Freiburg, Germany; 4 Comprehensive Cancer Center, Ruhr University Bochum (RUCCC), Bochum, Germany

**Keywords:** Endometrial hyperplasia, Endometrial cancer, Benign endometrial hyperplasia, Atypical endometrial hyperplasia, Endometrial intraepithelial neoplasia, Progestin therapy

## Abstract

Endometrial hyperplasia (EH) is the precursor lesion for endometrioid adenocarcinoma of the endometrium (EC), which represents the most common malignancy of the female reproductive tract in industrialized countries. The most important risk factor for the development of EH is chronic exposure to unopposed estrogen. Histopathologically, EH can be classified into EH without atypia (benign EH) and atypical EH/endometrial intraepithelial neoplasia (EIN). Clinical management ranges from surveillance or progestin therapy through to hysterectomy, depending on the risk of progression to or concomitant EC and the patient´s desire to preserve fertility. Multiple studies support the efficacy of progestins in treating both benign and atypical EH. This review summarizes the evidence base regarding risk factors and management of EH. Additionally, we performed a systematic literature search of the databases PubMed and Cochrane Controlled Trials register for studies analyzing the efficacy of progestin treatment in women with EH.

## Introduction

In industrialized countries, endometrial cancer (EC) represents the most common malignancy of the female reproductive tract. The precursor lesion for endometrioid adenocarcinoma of the endometrium, which comprises the majority of ECs, is endometrial hyperplasia (EH). EH is a noninvasive, abnormal proliferation of the endometrial lining of the uterus and associated with a significant risk of concurrent EC or progression to EC. The leading symptoms of EH are bleeding disorders in premenopausal women and vaginal bleeding in postmenopausal women. The most important risk factor is chronic exposure to unopposed estrogen. This narrative review aims to give an overview of the classification, risk factors, and management of EH. We searched the following databases: PubMed and Cochrane Controlled Trials register. We performed the final search in March 2021. Additionally, we performed a systematic literature search of the databases PubMed and Cochrane Controlled Trials register for studies analyzing the efficacy of progestin treatment in women with EH.

## Classification of EH and risk of progression to EC

Histologically, EH describes the abnormal proliferation of endometrial glands with a greater gland-to-stroma-ratio than healthy proliferative endometrium but without endometrial stromal invasion. Diagnosis should be based upon histological assessment of a tissue sample obtained by endometrial biopsy, curettage, or hysterectomy. The most widely used classification system for EH is the 2014 World Health Organization (WHO) Classification System which differentiates between:

- EH without atypia (benign EH) and
- atypical EH/endometrial intraepithelial neoplasia (EIN).

This distinction is particularly important because clinical management of the two conditions is different, depending on the presence or absence of nuclear atypia. Nuclear atypia is defined as nuclear enlargement with or without prominent nucleoli [1]. EH without atypia constitutes a benign lesion without significant somatic genetic changes caused by extensive exposure to estrogen that is not counterbalanced by the protective effects of progestins. If physiological progesterone levels are resumed or if therapeutic progestins are used, the hyperplastic changes regress and the endometrium becomes healthy again in the majority of cases [2]. EH without atypia seems to rarely progress to EC although evidence supporting this statement is poor. For example, in a case series published in 1985, benign EH only progressed to EC in two (1.6%) out of 122 patients. The first patient with simple hyperplasia developed atypical EH and subsequently EC 11 years after the first diagnosis of EH. The second patient had complex EH which progressed to EC 8.3 years after the initial diagnosis. Neither patient had received progestin treatment or died of their disease [3].

In a case–control study nested in a cohort of 7,947 women diagnosed with EH from 1970 to 2002, 138 women were diagnosed with EC, on average 6 years after the initial diagnosis of EH [4, 13]. The cumulative 20-year progression risk was less than 5% for EH without atypia but 28% for atypical EH. Specifically, for EH without atypia, the cumulative progression risk increased from 1.2% (95% CI, 0.6% to 1.9%) to 1.9% within 4 years (95% CI, 1.2% to 2.6%), to 4.6% within 9 years (95% CI, 3.3% to 5.8%) and up to 19 years after EH diagnosis. For women with atypical EH, the cumulative risk increased from 8.2% (95% CI, 1.3% to 14.6%) to 12.4% within 4 years (95% CI, 3.0% to 20.8%), to 27.5% within 9 years (95% CI, 8.6% to 42.5%) and up to 19 years after the diagnosis of EIN.

Atypical EH has many similarities with endometrioid EC at the molecular level [4]. Concurrent EC was found in 37% of women with atypical EH in a retrospective case series including 219 patients with EIN combined with a review of 31 published studies including a total of 2571 patients [5]. Other studies have documented a risk of concomitant EC in up to 50% of patients with atypical EH [6–9]. A high risk of concomitant EC among women with atypical EH is also consistent with the high risk of progression to EC observed among women with EH undergoing progestin treatment. This risk of progression to EC has been described to be between 15 and 28% [10–12]. For example, a retrospective cohort study investigated 242 women with atypical EH, of whom 74% received progestin therapy [12]. The rate of progression to EC was significantly higher without progestin treatment (101.4 versus 20.5 per 1000 woman-years). During a median follow-up of five years, 15% of all women with atypical EH developed EC. The risk for development of EC in women with atypical EH has been shown to be diminished approximately threefold to fivefold when treated with progestin [12]. Another important issue to be considered when assessing the concurrent or future risk of EC among women with atypical EH is the fact that it may be difficult to histopathologically distinguish EIN from EC. Illustrating these diagnostic difficulties, Trimble et al. found over- or underdiagnosis in almost every third specimen [13]. Specifically, independent gynecologic pathologists reviewed 289 endometrial biopsy specimens from women with a community diagnosis of atypical EH and found that in 25% the diagnosis was less severe than atypical EH, whereas in 29.1% EC was already present. This suggests a significant overlap between the different diagnoses of EH without atypia, atypical EH, and EC both in clinical practice as well as in the literature. Therefore, it is reasonable to treat atypical EH as the equivalent of early EC when counseling affected patients.

## Risk factors of EH

As its precursor lesion, the risk factors for developing EH are closely related to the well-known risk factors for endometrioid adenocarcinoma of the endometrium. The most important risk factor for both EH and EC is a chronic imbalance of estrogen and progestin in favor of estrogen [14–18]. Exposure to excessive estrogen without the protective effect of progestin can be endogenous or exogenous in nature. Other risk factors include genetic factors such as hereditary nonpolyposis colorectal cancer (HNPCC, or Lynch syndrome) and Cowden syndrome, which is rare and in most cases related to a mutation in the phosphatase and tensin homologue deleted on chromosome 10 (PTEN) gene, located on chromosome 10q23.31 [19–22].

## Endogenous estrogen exposure

Examples of excessive endogenous exposure to estrogen include obesity, chronic anovulation, early menarche, and late menopause as well as the presence of estrogen-secreting tumors. In obese women, a high amount of adipose tissue is closely associated with higher local and circulating levels of estradiol. This can be explained through various mechanisms. Firstly, an increase in adrenal secretory activity is often observed, which leads to an increase in androgen precursors. These precursors may then be converted to estradiol in peripheral tissues. Secondly, the conversion rate of androstenedione to estrone by the enzyme aromatase rises as this conversion primarily takes place in adipose tissue. Lastly, higher concentrations of estradiol can be found in obese patients as plasma levels of estradiol-binding sex hormone-binding globulin (SHBG) are typically depressed in this patient population [23]. These pathophysiological observations are consistent with clinical findings. For example, in a retrospective cohort study including 916 premenopausal women with abnormal uterine bleeding, patients with a body mass index of over 30 kg/m^2^ developed complex hyperplasia or EC 4 times more often than lean women (95% CI, 1.36–11.74) [24].

Chronic anovulation is another important risk factor for EH. When anovulation occurs, sex hormone production is not cyclical as in regularly ovulating premenopausal women. Without ovulation, estrogen levels still dominate without the opposing effect of progesterone produced by the corpus luteum after ovulation. This imbalance leads to a continued proliferation of the endometrium resulting in a higher risk of developing EH and eventually endometrioid EC. Common settings associated with anovulation include polycystic ovary syndrome (PCOS), hyperprolactinemia, and perimenopausal hormonal status. In 2009, a meta-analysis of four case–control studies including a total of 4056 women showed an almost threefold risk of developing EC for women with PCOS compared to the general population (OR 2.70, 95% CI, 1.00–7.29) [25]. In 2012, a subsequent meta-analysis of the same studies including another cross-sectional study, confirmed this risk elevation in PCOS patients (OR 2.89, 95% CI, 1.52–5.48) [26]. However, these results need to be interpreted with caution since other authors suspected that the risk increase was exaggerated due to risk factors commonly associated with PCOS such as obesity and diabetes and skewing of the risk estimates due to a potential selection bias in some of the studies [27, 28].

Hormone-secreting tumors can cause an endogenous sex hormone imbalance. For example, granulosa cell tumors represent potentially estrogen-secreting tumors of the ovary. Consequently, EH is diagnosed in 25% to 50% of women with granulosa cell tumors of the ovary [29, 30]. If EH is diagnosed in a patient without other known risk factors, estrogen-secreting tumors should therefore be excluded.

### Exogenous estrogen exposure

Estrogen therapy should always be supplemented with a progestin in women with an intact uterus. Unopposed estrogen therapy in women with an intact uterus increases the risk of developing EH and then EC in both premenopausal and in postmenopausal women. This is well illustrated in a meta-analysis including 45 randomized studies of hormone replacement therapy over a minimum period of 12 months in postmenopausal patients [31]. The authors documented a significantly increased risk of EH when women were treated with estrogen alone. The risk increased after 12 months of unopposed treatment with moderate or high-dose estrogen therapy (OR 8.4 and 10.7, respectively) and after 18 to 24 months of treatment with low-dose estrogen therapy (OR 2.4). Other studies have assessed the risk of developing EC in women treated with unopposed estrogens and found a 1.5- to tenfold relative risk increase [32–36].

Tamoxifen, which is one of the most important agents for endocrine treatment of hormone receptor-positive breast cancer, has been associated with an increased risk for developing EH and EC [37]. Several small studies concluded that tamoxifen-related ECs are mostly diagnosed at an early stage with an overall good prognosis [38–40]. In contrast, a nationwide case–control study conducted in the Netherlands including 309 patients with a diagnosis of EC after a history of breast cancer and 860 patients with a history of breast cancer but without EC reported a worse prognosis in long-term tamoxifen users due to a higher risk of EC with a higher tumor stage and a less favorable histology [41]. In this study, a longer duration of tamoxifen treatment was associated with a significantly increased risk of EC. The relative risks were 2.0 (1.2–3.2) for 2 to 5 years of tamoxifen and 6.9 (2.4–19.4) for at least 5 years of tamoxifen compared to no tamoxifen therapy. In addition, long-term use of tamoxifen (≥ 2 years) was associated with more stage III and IV ECs compared with non-users (17.4% and 5.4%, respectively, p < 0.01). However, in patients with hormone receptor positive breast cancer the benefits of endocrine therapy with tamoxifen clearly predominate in comparison with the increased risk of EC [41, 42]. A prophylactic use of tamoxifen in healthy patients, on the other hand, should be discussed critically in view of the available data. Two studies suggest that the elevated risk of EH and EC in tamoxifen users might be limited to postmenopausal women. In one of these studies, no significant difference in the development of endometrial abnormalities was observed in premenopausal patients treated with tamoxifen compared to women who received placebo. In contrast, the risk ratio among postmenopausal women was 4.01 (95% CI, 1.70 – 10.90) [43]. Another study found significantly more endometrial abnormalities in postmenopausal women treated with tamoxifen, while no differences in endometrial thickness, uterine volume, or histopathological results were reported in premenopausal patients treated with tamoxifen compared with controls [44]. This clinical aspect is important since tamoxifen can be replaced by aromatase inhibitors in postmenopausal breast cancer patients but still represents the standard of care for premenopausal breast cancer patients.

### Lynch syndrome

HNPCC or Lynch syndrome is a genetic disorder inherited in an autosomal dominant fashion. Due to mutations in DNA mismatch-repair (MMR) genes like MSH2, MLH1, MSH6 and PMS2 leading to microsatellite instability (MSI) patients with HNPCC have a lifetime risk of 40–60% for development of EC [45]. In a recent prospective cross-sectional study conducted in the United Kingdom 500 women with atypical EH or EC were tested for Lynch-Syndrome. In total, 16/500 participants had Lynch-Syndrome (3.2%, 95% CI 1.8% to 5.1%) and 11 more (2.5%) were tested positive for MMR variants of uncertain significance. The proportion of affected patients with Lynch-Syndrome (3.2%) in this study population was similar to colorectal cancer. Thus, the authors suggest unselected screening of atypical EH and EC for MSI [46]. EC is also diagnosed at a younger age in women with Lynch-Syndrome [47]. In a study including 69 women with Lynch-Syndrome-associated EC, 18% were diagnosed under 40 years, compared with a mean age of 60 years in women without Lynch-Syndrome [48]. Molecular alterations like MSI seem to take place early in the degenerative process. Studies suggest that determination of molecular changes like MSI in patients with atypical EH could help to identify early carcinogenesis and synchronous endometrial carcinoma [49, 50]. Experts recommend annual hysteroscopy and endometrial sampling in women with Lynch Syndrome beginning at age 35 [51]. This recommendation is supported by data from a prospective observational cohort study including 41 women with HNPCC comparing the accuracy of office hysteroscopy and endometrial sampling with transvaginal ultrasound alone. While both diagnostic methods had a similar specificity, the positive likelihood ratio was higher and the negative likelihood ratio lower in office hysteroscopy and endometrial sampling compared to transvaginal ultrasound [52]. However, larger studies are needed to confirm these findings.

## Management of EH

A number of different aspects should be considered in the treatment of EH. Depending on the histological features and the patient´s medical history, all established risk factors for progression to EC or the concurrent presence of the disease should be determined. In addition, a specialized gyneco-pathologist should be consulted given the diagnostic uncertainties of differentiating between EH, atypical EH, and EC. The 2014 WHO classification of EH for the two—EH without atypia (benign EH) and atypical EH—should be applied in order to guarantee comparability of histopathological data both in clinical practice and in academic studies. The presence or absence of nuclear atypia is the most important factor for appropriate therapy planning and monitoring. EH is mostly associated with excessive exposure to unopposed estrogen. It is therefore crucial to supplement any form of treatment by removing the source of excessive estrogen, for example, by encouraging obese patients to lose weight, stopping any type of unopposed estrogen therapy, treating anovulation (e.g., in PCOS patients or patients with hyperprolactinemia), or identifying and removing estrogen-secreting tumors. Additionally, it is important to consider the necessity of contraception and fertility issues in premenopausal EH patients, depending on their family planning status. The most important treatment options for EH can be divided into three subgroups:

1. Surveillance (watchful waiting)
2. Surgical treatment (hysterectomy ± bilateral salpingo-oophorectomy)
3. Progestin therapy

### Surveillance

Surveillance alone might be an option for patients with benign EH (EH without atypia) and desire to preserve fertility when progestin therapy is not an option. In these cases it is especially important to eliminate potential sources of excess estrogen. Follow-up endometrial sampling should be conducted to exclude progression to atypical EH or EC given the 5% risk of progression over 20 years [4].

### Surgical treatment

The risk of disease progression or simultaneous EC is high for atypical EH. Thus, hysterectomy should be recommended to most patients with atypical EH, especially to all postmenopausal patients and to premenopausal women who have completed their families. If surgery is not an option or if the patient wishes to preserve her fertility, progestin treatment with close follow-up might be considered as an alternative [2]. Total hysterectomy is the curative treatment of choice in patients with atypical EH eligible for surgery [53]. A supracervical approach should not be recommended as the cervix might be affected by precancerous lesions. Intraoperatively, the uterine specimen can be assessed for malignant disease by gross inspection and/or frozen section. However, the sensitivity of frozen section as a method for detecting EC during surgery is low at rates of only 73%–88% [54–56]. Of note, one study even reported a sensitivity of only 27% [57]. In addition, bilateral salpingo-oophorectomy should be considered in women with early EC given the high prevalence of ovarian cancer among young women with EC. For example, in a systematic literature review, 2904 cases of women with simultaneous EC and ovarian cancer (SEOC) were identified with 1035 (36%) of them being premenopausal or < 50 years of age [96]. The proportion of women with SEOC among all reported EC cases was 842/23,498 (3%). Microsatellite instability with mutations in mismatch repair genes compatible with HNPCC were identified in 40% of women analyzed. Thus, young women with EC have a high risk of synchronous ovarian cancer. In young women with EC, bilateral salpingo-oophorectomy or careful histological assessment of both ovaries is recommended in order to confirm or rule out SEOC [58]. HNPCC testing should be offered to all young women with EC. The option of bilateral salpingo-oophorectomy should be discussed individually with every patient taking into account potential risks of the additional procedure as well as long-term adverse effects and the risk of ovarian cancer.

### Progestin therapy

Multiple studies support the efficacy of progestins in treating both benign and atypical EH [59–64]. Progestin therapy is therefore the most widely used approach for treating women with EH. The progestin supply activates progesterone receptors, which leads to decidualization of the endometrial stroma and subsequently to endometrial thinning [2]. Especially in premenopausal women with benign EH and a desire to preserve fertility, progestin administration should be the therapy of first choice. When a high risk of concurrent EC is suspected, initial endometrial biopsy should be supplemented with a dilation and curettage procedure to exclude any malignancy. Progestin therapy is contraindicated in patients with thrombophilia, hormone receptor-positive breast cancer, liver failure, known allergy to progestins and during pregnancy.

Different types of progestins and different routes of administration have been proven to be effective in treating EH. While orally administered megestrol acetate and medroxyprogesterone acetate (MPA) were formerly the most widely used therapeutics, the levonorgestrel-releasing intrauterine device (LNG-IUD), which releases 20 μg of LNG over 24 h (LNG 52/5) for a period of 4 to 5 years, has replaced oral progestin therapies as current first-line therapy in many countries. Indeed, in patients with benign EH, regression to healthy endometrium has been reported in up to 90% upon treatment with the LNG 52/5 IUD [65, 66]. In women with atypical EH and/or early EC, treatment with the LNG 52/5 IUD has been shown to lead to complete regression in approximately 75–85% of patients [2]. Randomized trials reported that the LNG 52/5 IUD is more effective than orally administered progestins in the treatment of EH, while having fewer systemic side effects and providing effective contraception [67–69]. Through its local effect on the endometrium, the LNG 52/5 IUD evades absorption and metabolism by the intestinal flora and the liver, where progestin is associated with sedative effects [70, 71]. Other side effects of orally administered progestins include bloating, nausea, headaches, and mood swings or even depression. Irregular vaginal bleeding patterns, including spotting, represent another typical side effect of progestin treatment. This is true for both oral and intrauterine treatments. A meta-analysis of 34 observational studies found no difference in vaginal bleeding irregularities in women treated with oral progestins as compared with women who received the LNG 52/5 IUD [72].

Although potentially more effective, the LNG 52/5 IUD might not constitute an appropriate therapeutic approach for all patients with EH. Oral progestins might be a better option for women who are eager to have children, patients with IUD-related dysmenorrhea, or anatomic factors complicating IUD placement. When treating EH with oral progestins, patients should receive a continuous therapeutic dose over three to six months. Therapeutics include progestin monotherapies and combined estrogen-progestin drugs (oral contraceptives). As previously mentioned, the most common oral progestin monotherapeutics are megestrol acetate and MPA [64, 65]. The doses of MPA used in the literature depend on the type of EH and vary from 10 mg per day for benign EH to 600 mg per day in EIN [73]. Overall, in order to treat EIN progestin therapy should be administered continuously and in higher doses than for treatment of benign EH [66, 74–76]. Moreover, cyclic progestin therapies are more frequently associated with vaginal bleeding irregularities than continuous dosing.

However, these medications do not provide contraception. As progestin medications are contraindicated during pregnancy, additional birth control should be advised to premenopausal women using these therapies. To date, no high-quality data supporting the use of oral contraceptives in the treatment of EH are available, although the effect of progesterone dominates at the tissue site in combined estrogen-progestin drugs [77].

The therapeutic success of progestin therapy should be monitored by endometrial sampling on a 3-monthly or 6-monthly basis. Especially in women with atypical EH a thorough follow-up is mandatory. If regression to a healthy endometrium can be demonstrated histologically, premenopausal patients who would like to preserve fertility may end progestin therapy after 3 to 6 months and try to become pregnant. In fact, after successfully completing progestin therapy, some women with EH are able to conceive. A meta-analysis including 28 studies with a total of 1038 patients with atypical EH or early EC reported that 34% of patients conceived after progestin treatment (95% CI, 30–38%) and 20% of these women actually delivered a baby [73].

In postmenopausal women or premenopausal women without an immediate desire to become pregnant, progestin therapy should be continued as maintenance therapy with further follow-up via endometrial sampling, especially if vaginal bleeding abnormalities recur [78, 79]. If atypical EH persists or if EC develops despite continued progestin therapy, hysterectomy is recommended.

#### Systematic literature search “efficacy of progestin treatment in women with EH”

We performed a literature search of the databases PubMed and Cochrane Controlled Trials register (search date 30–06-2020) using the search terms: ((((("progestinic"[All Fields] OR "progestinics"[All Fields]) OR "progestins"[Pharmacological Action]) OR "progestins"[MeSH Terms]) OR "progestins"[All Fields]) OR "progestin"[All Fields]) AND (("endometrial hyperplasia"[MeSH Terms] OR ("endometrial"[All Fields] AND "hyperplasia"[All Fields])) OR "endometrial hyperplasia"[All Fields]) AND (((((("therapeutics"[MeSH Terms] OR "therapeutics"[All Fields]) OR "treatments"[All Fields]) OR "therapy"[MeSH Subheading]) OR "therapy"[All Fields]) OR "treatment"[All Fields]) OR "treatment s"[All Fields]). This search yielded 749 citations. We then restricted the search to the last 20 years (January 2000 to June 2020) resulting in 385 citations. The abstracts of these publications were screened and 33 of them were selected that included original data from retrospective or prospective comparisons of endocrine treatment options with progestins compared to placebo or no treatment. These studies were retrieved in full and a reference search was performed, yielding an additional 3 citations. In summary, we identified 7 systematic reviews and/or meta-analyses [65, 66, 72, 73, 80–82], 21 cohort studies [59, 61, 74–76, 78, 79, 83–96], 1 case–control study [60], 6 randomized controlled trials [97–102], and one case report [103]. Table 1 shows the study characteristics and results of the 6 randomized clinical trials with patient-specific data analyzing the efficacy of progestin treatment in women with EH. Table 2 shows the study characteristics and results of the 21 cohort studies with patient-specific data analyzing the efficacy of progestin treatment in women with EH.

**Table 1:** Patient characteristics and results of randomized controlled trials analyzing the efficacy of progestin treatment in women with EH

Author	Year	Study type	Number of patients	Population characteristics	Intervention	Regression of EH	Persistence/pro-gression of EH	Side effects
Dolapcioglu [97]	2013	RCT	104	simple EH (*n* = 61), atypical EH (*n* = 43)	Oral MPA (10 mg/day;* n* = 52) vs. LNG-IUD (*n* = 52) both for 3–6 months; 2-year follow-up	64% (MPA) vs. 100% (LNG-IUD) after 6 months	22/51 (43%) for MPA vs. 4/51 (8%) for LNG-IUD after 2 years; 50% (MPA) vs. 84% (LNG-IUD) after 3 months	–
Orbo [98]	2008	RCT	258	EH without further differentiation	LNG-IUD vs. oral progestin, observation for 6 months; follow-up for 24 months	LNG-IUD more effective vs. oral progestins and observation after 6 months (*p* = 0.001) and after follow-up	No case of EC during follow-up (56 to 108 months)	–
Ismail [99]	2013	RCT	90	EH without atypia	Cyclical MPA 10 mg/day vs. cyclical NETA 15 mg/day vs. LNG-IUD for 6 months	36.6% vs. 40% vs. 66.7%	No case of EC	–
Karimi-Zarchi [100]	2013	RCT	40	EH without atypia	Cyclical MPA 20 mg/day vs. LNG-IUD for 3 months	LNG-IUD more effective (*p* < 0.05)	–	Better satisfaction; less side effects with LNG-IUD
El-Behery [101]	2015	RCT	138	EH without further differentiation; diagnosed by ultrasound	Oral dydrogesterone vs. LNG-IUD for 6 months	LNG-IUD more effective (96% vs. 80%)	Recurrence rate lower with LNG-IUD (0% vs. 12%)	Patient satisfaction better with LNG-IUD despite more spotting
Orbo [102]; Update of [98]	2016	RCT	153	EH without further differentiation	LNG-IUD vs. oral progestin vs. observation for 6 months; follow-up for 24 months	–	Histological recurrence in 55/135 (41%) with CR; Recurrence rates similar in three therapy groups; recurrence dependent on menopausal status (*p* = 0.0005) and estrogen level (*p* = 0.0007)	–
*Pooled analysis*	–	RCT (*n* = 6 including one Update)	783	–	–	LNG-IUD with higher rates vs. oral progestins in all studies	LNG-IUD with lower rates of persistence/recurrence in 2 studies	Less side effects with LNG-IUD in 1 study; better patient satisfaction in 2 studies

**Table 2:** Patient characteristics and results of prospective and retrospective cohort studies analyzing the efficacy of progestin treatment in women with EH

Author	Year	Study type	Number of patients	Population characteristics	Intervention	Regression of EH	Persistence/progression of EH	Side effects
Reed [59]	2009	CS	185	Complex (*n* = 115) or atypical EH (*n* = 70) on independent pathology review	Progestin therapy (oral MPA or MGA or NETA) or no therapy	Complex EH: 59% (68/115) with progestins vs. 12% (14/115) with no therapy; Atypical EH: 54% (38/70) with progestins vs. 8% (6/70) with no therapy	28.4% with progestins vs. 30% with no therapy (complex EH); 26.9% with progestins vs. 66.7% with no therapy (atypical EH); EC G1 in 11/28 follow-up hysterectomies	–
Dhar [61]	2005	CS	4	Endometrioid EC, G1, PR positive	LNG-releasing IUD for at least 6 months	1/4	3/4	IUD expulsion (*n* = 3); emergency curettage (*n* = 1)
Wildemeersch [83]	2003	CS	12	Simple EH (*n* = 7), EH with atypia (*n* = 5)	LNG-releasing IUD (14 µg/d) for at least 12 months	12/12	One patient developed EC, G1, which regressed in consecutive biopsies	–
Mandel-Baum [84]	2020	CS	245	Atypical hyperplasia on in-house pathology report	Oral progestin therapy (*n* = 140 MGA; *n* = 28 MPA; *n* = 8 others) or LNG-IUD (*n* = 69) for at least 1 month	78.7% (LNG-IUD) vs. 46.7% (systemic progestins)	Progression to EC: 4.5% (LNG-IUD) vs. 15.7% (systemic progestins)	Morbidly obese women had higher benefit from LNG-IUD (HR 4.72; 95% CI 2.83–7.89) for CR)
Marra [75]	2014	CS	132	EH without atypia (simple or complex)	Oral progesterone in 2^nd^ half of menstrual cycle for 18 months or no treatment	95% vs. 75%, * p* = 0.05, for simple EH; 89% vs. 35%, * p* < 0.001, for complex EH	Regression rates were dose-dependent: 82%, 98%, and 100% for 100 mg, 200 mg and 300 mg	–
Simpson [76]	2014	CS	44	Atypical EH (*n* = 19), EC G1 (*n* = 25)	Oral progestin therapy (*n* = 140 MGA; *n* = 28 MPA; *n*= 8 others) or LNG-IUD (*n* = 69) for at least 1 month	24/44 (55%)	20/44 (45%); 13/44 with regression later recurred; 3/44 were up-staged	–
Park [79]	2013	CS	48	EC G1 with superficial myometrial invasion or EG G2/3 with no myometrial invasion	Oral progestin therapy (*n* = 14 MGA; *n* = 34 MPA) for a median of 6 months	37/48 (77%)	16/37 (43%)	Median time to CR 17 weeks; No mortality; 10 live births
Park [78]	2013	CS	33	Recurrence after progestin treatment for EC G1: atypical EH (*n* = 13), EC G1 (*n* = 20)	Oral progestin therapy (*n* = 3 MGA; MPA; *n* = 30) for a median of 6 months	28/33 (85%)	5/33 (15%)	No mortality
Wildemeersch [85]; Update of [83]	2007	CS	20	Simple EH (*n* = 12), EH with atypia (*n* = 8)	LNG-releasing IUD (20 µg/d) for at 14–90 months	11/12	1/12 had persisting benign EH	–
Yang [86]	2019	CS	160	atypical EH (*n* = 120), EC stage I without myometrial invasion (*n* = 40)	Hysteroscopic resection + oral progestin therapy until CR	148/160 (93%)	4/160 (2%)	15 of 60 attempting pregnancy became pregnant
Pal [74]	2018	CS	32	atypical EH (*n* = 17), EC G1/2, stage I (*n* = 15)	LNG-IUD for 6 months	80% (atypical EH) vs. 67% (EC G1) vs. 75% (EC G2)	3/32	1/5 became pregnant and delivered
Scarselli [87]	2010	CS	34	EH without atypia (*n* = 30), atypical EH (*n* = 4)	LNG-IUD (20 µg/day) for 5 years (range 12–60 months)	32/34	2/32 persistence; after mean follow-up of 17 years 9 had hysterectomy with EH in 5/9 cases	
Buttini [88]	2009	CS	57	EH without atypia (*n* = 41), EH with atypia (*n* = 16)	LNG-IUD (*n* = 26), oral progestin (*n* = 10), hysterectomy (*n* = 21)	21/26 (LNG-IUD) vs. 9/10 (progestin)	2/32 persistence; 0/57 developed EC	1 LNG-IUD removed for side effects
Varma [89]	2008	CS	105	EH without atypia (*n* = 96), EH with atypia (*n* = 9)	LNG-IUD for 2 years	96% (90/94) after 1 year; 90% (94/105) after 2 years; 88/96 (92%) for EH without atypia and 6/9 (67%) for EH with atypia	1 case of EC	–
Gallos [90]	2013	CS	344	Complex EH without atypia or EH with atypia	Oral progestins (*n* = 94) or LNG-IUD (*n* = 250)	95% (237/250) for LNG-IUD vs. 84% (79/94) for oral progestins (OR 3.04; 95% CI 1.4–6.8)	8 cases of EC	Hysterectomy rates were 55/250 (22%) for LNG-IUD vs. 35/94 (37%) for oral progestins
Gallos [91]; Update of [90]	2013	CS	219	Complex EH without atypia or EH with atypia who achieved CR after progestin treatment	Oral progestins or LNG-IUD	–	21/153 (14%) for LNG-IUD vs. 20/66 (30%) for oral progestin; 2 cases of EC	Hysterectomy rates lower for LNG-IUD (20% vs. 32%)
Cholakian [92]	2016	CS	60	EH with atypia (*n* = 25); EC G1 (*n* = 35)	MGA (*n* = 42); MPA (*n* = 11); LNG-IUD (*n* = 22); multiple regimens possible	–	–	Median weight change greater for MGA vs. LNG-IUD (+ 2.9 vs. + 0.05 kg); BMI < 35 gained more weight vs. BMI ≥ 35 (+ 2.3 vs. − 0.7 kg/month); for BMI ≥ 35, MGA had more weight gain than LNG-IUD (+ 2.2 vs. − 5.4 kg)
Kim [93]	2016	CS	75	EH without atypia (*n* = 60); EH with atypia (*n* = 15)	LNG-IUD for 12 months	95% (36/38) after 12 months	1 case with residual EH	–
Marnach [94]	2017	CS	94	Endometrial intraepithelial neoplasia	LNG-IUD	87% (no atypia); 62% (with atypia); 22% (adenocarcinoma)	–	–
Haoula [95]	2011	CS	51	EH without atypia (*n* = 32); EH with atypia (*n* = 19)	LNG-IUD for 12 months	97% (31/32) for EH without atypia after 24 months; 84% (16/19) for atypical EH	2 cases of persistence	–
Kim [96]	2013	CS	16	EC G1, < 2 cm	LNG-IUD + oral MPA (500 mg/day) for 3 months	88% (14/16); median time to CR 9.8 months	No case of progression	No treatment-related complications
Pooled analysis	**-**	CS (*n* = 21 including two Updates)	1087	–	–	LNG-IUD with higher rates vs. oral progestins in 7 studies	Progression to EC lower with LNG-IUD in 2 studies; regression rates dose-dependent with oral progestins in 1 study	Hysterectomy rates lower for LNG-IUD in 2 studies; more weight gain for MPA/MGA than LNG-IUD in 1 study

EH, endometrial hyperplasia; CS, cohort study; MPA, medroxyprogesterone acetate; MGA, megestrol acetate; NETA, norethisterone acetate; EC, endometrial cancer; G1, grading 1; LNG, levonorgestrel; IUD, intrauterine device; PR, progesterone receptor; RCT, randomized controlled trial; BMI, body mass index; CR, complete response.

EH, endometrial hyperplasia; CS, cohort study; MPA, medroxyprogesterone acetate; MGA, megestrol acetate; NETA, norethisterone acetate; EC, endometrial cancer; G1, grading 1; LNG, levonorgestrel; IUD, intrauterine device; PR, progesterone receptor; RCT, randomized controlled trial; BMI, body mass index; CR, complete response.

In addition to the RCTs and CS listed in Tables 1 and 2, we identified 1 case–control study [60] and 1 case report [103]. In the case–control study, Montz et al. assessed the feasibility of using a. LNG-IUD to treat presumed FIGO stage IA, grade 1 EC in women at high risk for perioperative complications, i.e. American Society of Anesthesiologists class III or IV. Twelve patients have been followed up to 36 months; results of biopsies were negative in 7 of 11 at 6 months and 6 of 8 at 12 months. No IUD-related complications, except for expulsion, occurred. Sixteen complications (one fatal) occurred in 9 of the 15 control patients who underwent surgery. The authors conclude that LNG-IUD is a feasible treatment with special relevance for patients with early EC and a high risk of surgical complications. In the case report, Kresowik et al. report a patient with atypical endometrial hyperplasia who developed an adenocarcinoma of the endometrium after 6 months of treatment with a LNG-IUD. The authors advocate caution when using this therapy and recommend rigorous and in-depth shared decision making.

#### Systematic reviews of progestin treatment for EH or early EC

In a systematic review and meta-analysis of 7 randomized trials with 766 patients, Abu Hashim et al. found that the LNG-IUD achieved a higher regression rate than oral progestins after 3, 6, 12, and 24 months (OR, 7.46; 95% CI, 2.55–21.78). Subgroup analysis showed that the effect of treatment with the LNG-IUD was superior for both simple and complex EH [65]. In another systematic review and meta-analysis including 24 observational studies and 1001 patients, Gallos et al. confirmed the superiority of the LNG-IUD to oral progestins both for complex EH (pooled regression rate, 66% vs 92%; *p* < 0.01) and atypical EH (pooled regression rate, 69% vs 90%; *p* = 0.03) [66]. In a second meta-analysis of 34 observational studies, Gallos et al. investigated the efficacy of conservative treatment in women with early EC (408 patients) or atypical EH (151 patients) [72]. Endocrine treatment achieved a pooled regression rate of 76%, a relapse rate of 41%, and a live birth rate of 28%. Twenty women were diagnosed with ovarian cancer (concurrent or metastatic) during follow-up (4%) and 10 progressed to higher than stage I EC (2%), among whom 2 women died. These data show that endocrine treatment of early stage EC is feasible, but is associated with a significant morbidity and mortality, which can be estimated at around 1 in 200. Wei et al. found a similar efficacy of progestins in women with EC G1 or atypical EH summarizing 28 studies with 1038 patients [73]. Specifically, women with EC G1 or atypical EH had a pooled rate of complete regression (CR) of 71%. Although 34% of women became pregnant, only 20% of them delivered live newborns. The CR rate for women using progestin plus IUD was higher than for IUD alone (87% versus 76%).

A Cochrane meta-analysis compared oral and intrauterine progestins for atypical EH and included only one randomized trial in which a levonorgestrel-releasing intrauterine device (LNG-IUD) was found to be superior to oral medroxyprogesterone acetate (MPA) [82]. In a more recent systematic review and meta-analysis, this finding was confirmed by Yuk et al. [75]. That meta-analysis included five RCTs with 377 patients. Here, the regression rate in women using the LNG-IUD was higher than for oral MPA among lean women (relative risk [RR] 1.41; 95% CI 1.23–1.62), whereas the two regression rates, for LNG-IUD and oral MPA, were similar among obese women (RR 1.03; 95% CI 0.94–1.13). The LNG-IUD treatment was also superior in nonatypical EH (RR 1.36; 95% CI 1.07–1.73) and mixed EH (atypical and non-atypical) (RR 1.44; 95% CI 1.21–1.71).

There is weak evidence from a meta-analysis of two RCTs with 59 patients that metformin may be equally as effective as megestrol acetate (MGA) for the treatment of EH [76].

## Conclusion

In summary, the available data from individual studies (Table 1) and meta-analyses clearly show that progestins are a safe and very effective treatment in patients with EH without atypia. In these patients, oral progestins achieve regression rates of around 85% and the LNG-IUD achieves regression rates of up to 100%. In contrast, the efficacy of progestins is significantly lower in patients with atypical EH or EC G1. In these patients, regression rates between 70 and 85% may be expected and recurrence rates are high at to 40%. Treatment with the LNG-IUD seems to be more effective than oral progestins in patients with atypical EH or EC G1. The rates of live births after treatment are low at around 20%. Obese women gain more benefit from progestin treatment due to the higher relative background risk, but the treatment efficacy of progestins is higher in lean women. Additionally and notably, endocrine treatment of atypical EH or EC G1 carries a clinically relevant risk of mortality, which can be estimated as 1 in 200. Therefore, patients with atypical EH or EC G1 attempting treatment with progestins should be made aware of the low live-birth rates, the high recurrence risk, and the mortality associated with this kind of treatment. The standard of care for patients with atypical EH or EC G1 remains total hysterectomy.

## References

[1] Kurman RJ, Norris HJ (1982). Evaluation of criteria for distinguishing atypical endometrial hyperplasia from well-differentiated carcinoma. Cancer.

[2] Gunderson CC, Fader AN, Carson KA, Bristow RE (2012). Oncologic and reproductive outcomes with progestin therapy in women with endometrial hyperplasia and grade 1 adenocarcinoma: a systematic review. Gynecol Oncol.

[3] Kurman RJ, Kaminski PF, Norris HJ: The behavior of endometrial hyperplasia. A long-term study of "untreated" hyperplasia in 170 patients. *Cancer* 1985, 56(2):403–412. 10.1002/1097-0142(19850715)56:2<403::aid-cncr2820560233>3.0.co;2-x 4005805

[4] Cancer Genome Atlas Research N, Kandoth C, Schultz N, Cherniack AD, Akbani R, Liu Y, Shen H, Robertson AG, Pashtan I, Shen R *et al*: Integrated genomic characterization of endometrial carcinoma. *Nature* 2013, 497(7447):67–73. 10.1038/nature12113 PMC3704730 23636398

[5] Rakha E, Wong SC, Soomro I, Chaudry Z, Sharma A, Deen S, Chan S, Abu J, Nunns D, Williamson K (2012). Clinical outcome of atypical endometrial hyperplasia diagnosed on an endometrial biopsy: institutional experience and review of literature. Am J Surg Pathol.

[6] Silverberg SG (2000). Problems in the differential diagnosis of endometrial hyperplasia and carcinoma. Mod Pathol.

[7] Soslow RA (2006). Problems with the current diagnostic approach to complex atypical endometrial hyperplasia. Cancer.

[8] Shutter J, Wright TC (2005). Prevalence of underlying adenocarcinoma in women with atypical endometrial hyperplasia. Int J Gynecol Pathol.

[9] Valenzuela P, Sanz JM, Keller J (2003). Atypical endometrial hyperplasia: grounds for possible misdiagnosis of endometrial adenocarcinoma. Gynecol Obstet Invest.

[10] Vereide AB, Arnes M, Straume B, Maltau JM, Orbo A (2003). Nuclear morphometric changes and therapy monitoring in patients with endometrial hyperplasia: a study comparing effects of intrauterine levonorgestrel and systemic medroxyprogesterone. Gynecol Oncol.

[11] Lacey JV, Sherman ME, Rush BB, Ronnett BM, Ioffe OB, Duggan MA, Glass AG, Richesson DA, Chatterjee N, Langholz B (2010). Absolute risk of endometrial carcinoma during 20-year follow-up among women with endometrial hyperplasia. Journal of clinical oncology : official journal of the American Society of Clinical Oncology.

[12] Reed SD, Newton KM, Garcia RL, Allison KH, Voigt LF, Jordan CD, Epplein M, Swisher E, Upson K, Ehrlich KJ (2010). Complex hyperplasia with and without atypia: clinical outcomes and implications of progestin therapy. Obstet Gynecol.

[13] Trimble CL, Kauderer J, Zaino R, Silverberg S, Lim PC, Burke JJ, Alberts D, Curtin J (2006). Concurrent endometrial carcinoma in women with a biopsy diagnosis of atypical endometrial hyperplasia: a Gynecologic Oncology Group study. Cancer.

[14] Brinton LA, Berman ML, Mortel R, Twiggs LB, Barrett RJ, Wilbanks GD, Lannom L, Hoover RN (1992). Reproductive, menstrual, and medical risk factors for endometrial cancer: results from a case-control study. Am J Obstet Gynecol.

[15] Zeleniuch-Jacquotte A, Akhmedkhanov A, Kato I, Koenig KL, Shore RE, Kim MY, Levitz M, Mittal KR, Raju U, Banerjee S (2001). Postmenopausal endogenous oestrogens and risk of endometrial cancer: results of a prospective study. Br J Cancer.

[16] Lukanova A, Lundin E, Micheli A, Arslan A, Ferrari P, Rinaldi S, Krogh V, Lenner P, Shore RE, Biessy C (2004). Circulating levels of sex steroid hormones and risk of endometrial cancer in postmenopausal women. Int J Cancer.

[17] Nyholm HC, Nielsen AL, Lyndrup J, Dreisler A, Hagen C, Haug E (1993). Plasma oestrogens in postmenopausal women with endometrial cancer. Br J Obstet Gynaecol.

[18] Potischman N, Hoover RN, Brinton LA, Siiteri P, Dorgan JF, Swanson CA, Berman ML, Mortel R, Twiggs LB, Barrett RJ (1996). Case-control study of endogenous steroid hormones and endometrial cancer. J Natl Cancer Inst.

[19] Tzortzatos G, Andersson E, Soller M, Askmalm MS, Zagoras T, Georgii-Hemming P, Lindblom A, Tham E, Mints M (2015). The gynecological surveillance of women with Lynch syndrome in Sweden. Gynecol Oncol.

[20] Pilarski R, Stephens JA, Noss R, Fisher JL, Prior TW (2011). Predicting PTEN mutations: an evaluation of Cowden syndrome and Bannayan-Riley-Ruvalcaba syndrome clinical features. J Med Genet.

[21] Riegert-Johnson DL, Gleeson FC, Roberts M, Tholen K, Youngborg L, Bullock M, Boardman LA (2010). Cancer and Lhermitte-Duclos disease are common in Cowden syndrome patients. Hered Cancer Clin Pract.

[22] Heald B, Mester J, Rybicki L, Orloff MS, Burke CA, Eng C (2010). Frequent gastrointestinal polyps and colorectal adenocarcinomas in a prospective series of PTEN mutation carriers. Gastroenterology.

[23] Siiteri PK (1987). Adipose tissue as a source of hormones. Am J Clin Nutr.

[24] Wise MR, Gill P, Lensen S, Thompson JM, Farquhar CM: Body mass index trumps age in decision for endometrial biopsy: cohort study of symptomatic premenopausal women. *American journal of obstetrics and gynecology* 2016, 215(5):598 e591–598 e598. 10.1016/j.ajog.2016.06.006 27287687

[25] Chittenden BG, Fullerton G, Maheshwari A, Bhattacharya S (2009). Polycystic ovary syndrome and the risk of gynaecological cancer: a systematic review. Reprod Biomed Online.

[26] Haoula Z, Salman M, Atiomo W (2012). Evaluating the association between endometrial cancer and polycystic ovary syndrome. Hum Reprod.

[27] Barry JA, Azizia MM, Hardiman PJ (2014). Risk of endometrial, ovarian and breast cancer in women with polycystic ovary syndrome: a systematic review and meta-analysis. Hum Reprod Update.

[28] Rosen MW, Tasset J, Kobernik EK, Smith YR, Johnston C, Quint EH (2019). Risk factors for endometrial cancer or hyperplasia in adolescents and women 25 years old or younger. J Pediatr Adolesc Gynecol.

[29] Schumer ST, Cannistra SA (2003). Granulosa cell tumor of the ovary. Journal of clinical oncology : official journal of the American Society of Clinical Oncology.

[30] Zanagnolo V, Pasinetti B, Sartori E (2004). Clinical review of 63 cases of sex cord stromal tumors. Eur J Gynaecol Oncol.

[31] Furness S, Roberts H, Marjoribanks J, Lethaby A, Hickey M, Farquhar C: Hormone therapy in postmenopausal women and risk of endometrial hyperplasia. *Cochrane Database Syst Rev* 2009(2):CD000402. 10.1002/14651858.CD000402.pub3 19370558

[32] Henderson BE (1989). The cancer question: an overview of recent epidemiologic and retrospective data. Am J Obstet Gynecol.

[33] Persson I, Adami HO, Bergkvist L, Lindgren A, Pettersson B, Hoover R, Schairer C (1989). Risk of endometrial cancer after treatment with oestrogens alone or in conjunction with progestogens: results of a prospective study. BMJ.

[34] Beral V, Bull D, Reeves G (2005). Million Women Study C: Endometrial cancer and hormone-replacement therapy in the Million Women Study. Lancet (London, England).

[35] Weiderpass E, Adami HO, Baron JA, Magnusson C, Bergstrom R, Lindgren A, Correia N, Persson I (1999). Risk of endometrial cancer following estrogen replacement with and without progestins. J Natl Cancer Inst.

[36] Strom BL, Schinnar R, Weber AL, Bunin G, Berlin JA, Baumgarten M, DeMichele A, Rubin SC, Berlin M, Troxel AB (2006). Case-control study of postmenopausal hormone replacement therapy and endometrial cancer. Am J Epidemiol.

[37] Committee Opinion No (2014). 601: Tamoxifen and uterine cancer. Obstet Gynecol.

[38] Fisher B, Costantino JP, Redmond CK, Fisher ER, Wickerham DL, Cronin WM (1994). Endometrial cancer in tamoxifen-treated breast cancer patients: findings from the National Surgical Adjuvant Breast and Bowel Project (NSABP) B-14. J Natl Cancer Inst.

[39] Jordan VC, Morrow M: Should clinicians be concerned about the carcinogenic potential of tamoxifen? *European journal of cancer (Oxford, England : 1990)* 1994, 30A(11):1714–1721. 10.1016/0959-8049(94)00349-a 7833150

[40] Barakat RR, Wong G, Curtin JP, Vlamis V, Hoskins WJ (1994). Tamoxifen use in breast cancer patients who subsequently develop corpus cancer is not associated with a higher incidence of adverse histologic features. Gynecol Oncol.

[41] Bergman L, Beelen ML, Gallee MP, Hollema H, Benraadt J, van Leeuwen FE: Risk and prognosis of endometrial cancer after tamoxifen for breast cancer. Comprehensive Cancer Centres' ALERT Group. Assessment of Liver and Endometrial cancer Risk following Tamoxifen. *Lancet (London, England)* 2000, 356(9233):881–887. 10.1016/s0140-6736(00)02677-5 11036892

[42] Tamoxifen for early breast cancer: an overview of the randomised trials. Early Breast Cancer Trialists' Collaborative Group. *Lancet (London, England)* 1998, 351(9114):1451–1467. 9605801

[43] Fisher B, Costantino JP, Wickerham DL, Redmond CK, Kavanah M, Cronin WM, Vogel V, Robidoux A, Dimitrov N, Atkins J (1998). Tamoxifen for prevention of breast cancer: report of the National Surgical Adjuvant Breast and Bowel Project P-1 Study. J Natl Cancer Inst.

[44] Cheng WF, Lin HH, Torng PL, Huang SC (1997). Comparison of endometrial changes among symptomatic tamoxifen-treated and nontreated premenopausal and postmenopausal breast cancer patients. Gynecol Oncol.

[45] Lancaster JM, Powell CB, Chen LM, Richardson DL (2015). Committee SGOCP: Society of Gynecologic Oncology statement on risk assessment for inherited gynecologic cancer predispositions. Gynecol Oncol.

[46] Ryan NAJ, McMahon R, Tobi S, Snowsill T, Esquibel S, Wallace AJ, Bunstone S, Bowers N, Mosneag IE, Kitson SJ *et al*: The proportion of endometrial tumours associated with Lynch syndrome (PETALS): A prospective cross-sectional study. *PLoS Med* 2020, 17(9):e1003263. 10.1371/journal.pmed.1003263 PMC7497985 32941469

[47] Post CCB, Stelloo E, Smit V, Ruano D, Tops CM, Vermij L, Rutten TA, Jurgenliemk-Schulz IM, Lutgens L, Jobsen JJ *et al*: Prevalence and Prognosis of Lynch Syndrome and Sporadic Mismatch Repair Deficiency in Endometrial Cancer. *Journal of the National Cancer Institute* 2021. 10.1093/jnci/djab029 PMC8418420 33693762

[48] Schmeler KM, Lynch HT, Chen LM, Munsell MF, Soliman PT, Clark MB, Daniels MS, White KG, Boyd-Rogers SG, Conrad PG (2006). Prophylactic surgery to reduce the risk of gynecologic cancers in the Lynch syndrome. N Engl J Med.

[49] Zauber P, Denehy TR, Taylor RR, Ongcapin EH, Marotta S, Sabbath-Solitare M (2015). Strong correlation between molecular changes in endometrial carcinomas and concomitant hyperplasia. International journal of gynecological cancer : official journal of the International Gynecological Cancer Society.

[50] Horowitz N, Pinto K, Mutch DG, Herzog TJ, Rader JS, Gibb R, Bocker-Edmonston T, Goodfellow PJ (2002). Microsatellite instability, MLH1 promoter methylation, and loss of mismatch repair in endometrial cancer and concomitant atypical hyperplasia. Gynecol Oncol.

[51] Colombo N, Creutzberg C, Amant F, Bosse T, Gonzalez-Martin A, Ledermann J, Marth C, Nout R, Querleu D, Mirza MR *et al*: ESMO-ESGO-ESTRO Consensus Conference on Endometrial Cancer: diagnosis, treatment and follow-up. *Annals of oncology : official journal of the European Society for Medical Oncology / ESMO* 2016, 27(1):16–41. 10.1093/annonc/mdv484 26634381

[52] Manchanda R, Saridogan E, Abdelraheim A, Johnson M, Rosenthal AN, Benjamin E, Brunell C, Side L, Gessler S, Jacobs I (2012). Annual outpatient hysteroscopy and endometrial sampling (OHES) in HNPCC/Lynch syndrome (LS). Arch Gynecol Obstet.

[53] Trimble CL, Method M, Leitao M, Lu K, Ioffe O, Hampton M, Higgins R, Zaino R, Mutter GL (2012). Society of Gynecologic Oncology Clinical Practice C: Management of endometrial precancers. Obstet Gynecol.

[54] Attard Montalto S, Coutts M, Devaja O, Summers J, Jyothirmayi R, Papadopoulos A (2008). Accuracy of frozen section diagnosis at surgery in pre- malignant and malignant lesions of the endometrium. Eur J Gynaecol Oncol.

[55] Salman MC, Usubutun A, Dogan NU, Yuce K (2009). The accuracy of frozen section analysis at hysterectomy in patients with atypical endometrial hyperplasia. Clin Exp Obstet Gynecol.

[56] Morotti M, Menada MV, Moioli M, Sala P, Maffeo I, Abete L, Fulcheri E, Menoni S, Venturini P, Papadia A (2012). Frozen section pathology at time of hysterectomy accurately predicts endometrial cancer in patients with preoperative diagnosis of atypical endometrial hyperplasia. Gynecol Oncol.

[57] Indermaur MD, Shoup B, Tebes S, Lancaster JM (2007). The accuracy of frozen pathology at time of hysterectomy in patients with complex atypical hyperplasia on preoperative biopsy. Am J Obstet Gynecol.

[58] Weng CH, Wu RC, Chen SJ, Chen HC, Tan KT, Lee YS, Huang SS, Yang LY, Wang CJ, Chou HH *et al*: Molecular evidence for a clonal relationship between synchronous uterine endometrioid carcinoma and ovarian clear cell carcinoma: a new example of "precursor escape"? *Journal of molecular medicine (Berlin, Germany)* 2021. 10.1007/s00109-021-02064-4 33768299

[59] Reed SD, Voigt LF, Newton KM, Garcia RH, Allison HK, Epplein M, Jordan D, Swisher E, Weiss NS (2009). Progestin therapy of complex endometrial hyperplasia with and without atypia. Obstet Gynecol.

[60] Montz FJ, Bristow RE, Bovicelli A, Tomacruz R, Kurman RJ (2002). Intrauterine progesterone treatment of early endometrial cancer. Am J Obstet Gynecol.

[61] Dhar KK, NeedhiRajan T, Koslowski M, Woolas RP (2005). Is levonorgestrel intrauterine system effective for treatment of early endometrial cancer? Report of four cases and review of the literature. Gynecol Oncol.

[62] McCawley GM, Ferriss JS, Geffel D, Northup CJ, Modesitt SC (2009). Cancer in obese women: potential protective impact of bariatric surgery. J Am Coll Surg.

[63] Alonso S, Castellanos T, Lapuente F, Chiva L (2015). Hysteroscopic surgery for conservative management in endometrial cancer: a review of the literature. Ecancermedicalscience.

[64] Wildemeersch D, Dhont M (2003). Treatment of nonatypical and atypical endometrial hyperplasia with a levonorgestrel-releasing intrauterine system. Am J Obstet Gynecol.

[65] Abu Hashim H, Ghayaty E, El Rakhawy M (2015). Levonorgestrel-releasing intrauterine system vs oral progestins for non-atypical endometrial hyperplasia: a systematic review and metaanalysis of randomized trials. Am J Obstet Gynecol.

[66] Gallos ID, Shehmar M, Thangaratinam S, Papapostolou TK, Coomarasamy A, Gupta JK: Oral progestogens vs levonorgestrel-releasing intrauterine system for endometrial hyperplasia: a systematic review and metaanalysis. *American journal of obstetrics and gynecology* 2010, 203(6):547 e541–510. 10.1016/j.ajog.2010.07.037 20934679

[67] Ferenczy A, Gelfand M (1989). The biologic significance of cytologic atypia in progestogen-treated endometrial hyperplasia. Am J Obstet Gynecol.

[68] Affinito P, Di Carlo C, Di Mauro P, Napolitano V, Nappi C (1994). Endometrial hyperplasia: efficacy of a new treatment with a vaginal cream containing natural micronized progesterone. Maturitas.

[69] Perino A, Quartararo P, Catinella E, Genova G, Cittadini E (1987). Treatment of endometrial hyperplasia with levonorgestrel releasing intrauterine devices. Acta Eur Fertil.

[70] Arafat ES, Hargrove JT, Maxson WS, Desiderio DM, Wentz AC, Andersen RN (1988). Sedative and hypnotic effects of oral administration of micronized progesterone may be mediated through its metabolites. Am J Obstet Gynecol.

[71] Bourgain C, Devroey P, Van Waesberghe L, Smitz J, Van Steirteghem AC (1990). Effects of natural progesterone on the morphology of the endometrium in patients with primary ovarian failure. Hum Reprod.

[72] Gallos ID, Yap J, Rajkhowa M, Luesley DM, Coomarasamy A, Gupta JK: Regression, relapse, and live birth rates with fertility-sparing therapy for endometrial cancer and atypical complex endometrial hyperplasia: a systematic review and metaanalysis. *American journal of obstetrics and gynecology* 2012, 207(4):266 e261–212. 10.1016/j.ajog.2012.08.011 23021687

[73] Wei J, Zhang W, Feng L, Gao W: Comparison of fertility-sparing treatments in patients with early endometrial cancer and atypical complex hyperplasia: A meta-analysis and systematic review. *Medicine (Baltimore)* 2017, 96(37):e8034. 10.1097/MD.0000000000008034 PMC5604661 28906392

[74] Pal N, Broaddus RR, Urbauer DL, Balakrishnan N, Milbourne A, Schmeler KM, Meyer LA, Soliman PT, Lu KH, Ramirez PT (2018). Treatment of low-risk endometrial cancer and complex atypical hyperplasia with the levonorgestrel-releasing intrauterine device. Obstet Gynecol.

[75] Marra C, Penati C, Ferrari L, Cantu MG, Bargossi L, Fruscio R (2014). Treatment of simple and complex endometrial non-atypical hyperplasia with natural progesterone: response rate to different doses. Gynecol Endocrinol.

[76] Simpson AN, Feigenberg T, Clarke BA, Gien LT, Ismiil N, Laframboise S, Massey C, Ferguson SE (2014). Fertility sparing treatment of complex atypical hyperplasia and low grade endometrial cancer using oral progestin. Gynecol Oncol.

[77] Schindler AE (2013). Non-contraceptive benefits of oral hormonal contraceptives. Int J Endocrinol Metab.

[78] Park JY, Lee SH, Seong SJ, Kim DY, Kim TJ, Kim JW, Kim JH, Kim YM, Kim YT, Bae DS (2013). Progestin re-treatment in patients with recurrent endometrial adenocarcinoma after successful fertility-sparing management using progestin. Gynecol Oncol.

[79] Park JY, Kim DY, Kim TJ, Kim JW, Kim JH, Kim YM, Kim YT, Bae DS, Nam JH (2013). Hormonal therapy for women with stage IA endometrial cancer of all grades. Obstet Gynecol.

[80] Yuk JS, Song JY, Lee JH, Park WI, Ahn HS, Kim HJ (2017). Levonorgestrel-releasing intrauterine systems versus oral cyclic medroxyprogesterone acetate in endometrial hyperplasia therapy: a meta-analysis. Ann Surg Oncol.

[81] Clement NS, Oliver TR, Shiwani H, Sanner JR, Mulvaney CA, Atiomo W: Metformin for endometrial hyperplasia. *Cochrane Database Syst Rev* 2017, 10:CD012214. 10.1002/14651858.CD012214.pub2 PMC6485333 29077194

[82] Luo L, Luo B, Zheng Y, Zhang H, Li J, Sidell N: Oral and intrauterine progestogens for atypical endometrial hyperplasia. *Cochrane Database Syst Rev* 2018, 12:CD009458. 10.1002/14651858.CD009458.pub3 PMC6517239 30521671

[83] Wildemeersch D, Batar I, Affandi B, Andrade A, Shangchun W, Jing H, Xiaoming C (2003). The 'frameless' intrauterine system for long-term, reversible contraception: a review of 15 years of clinical experience. J Obstet Gynaecol Res.

[84] Mandelbaum RS, Ciccone MA, Nusbaum DJ, Khoshchehreh M, Purswani H, Morocco EB, Smith MB, Matsuzaki S, Dancz CE, Ozel B *et al*: Progestin therapy for obese women with complex atypical hyperplasia: levonorgestrel-releasing intrauterine device vs systemic therapy. *American journal of obstetrics and gynecology* 2020, 223(1):103 e101–103 e113. 10.1016/j.ajog.2019.12.273 PMC7751571 31978437

[85] Wildemeersch D, Janssens D, Pylyser K, De Wever N, Verbeeck G, Dhont M, Tjalma W (2007). Management of patients with non-atypical and atypical endometrial hyperplasia with a levonorgestrel-releasing intrauterine system: long-term follow-up. Maturitas.

[86] Yang B, Xu Y, Zhu Q, Xie L, Shan W, Ning C, Xie B, Shi Y, Luo X, Zhang H (2019). Treatment efficiency of comprehensive hysteroscopic evaluation and lesion resection combined with progestin therapy in young women with endometrial atypical hyperplasia and endometrial cancer. Gynecol Oncol.

[87] Scarselli G, Bargelli G, Taddei GL, Marchionni M, Peruzzi E, Pieralli A, Mattei A, Buccoliero AM, Fambrini M (2011). Levonorgestrel-releasing intrauterine system (LNG-IUS) as an effective treatment option for endometrial hyperplasia: a 15-year follow-up study. Fertil Steril.

[88] Buttini MJ, Jordan SJ, Webb PM (2009). The effect of the levonorgestrel releasing intrauterine system on endometrial hyperplasia: an Australian study and systematic review. Aust N Z J Obstet Gynaecol.

[89] Varma R, Soneja H, Bhatia K, Ganesan R, Rollason T, Clark TJ, Gupta JK (2008). The effectiveness of a levonorgestrel-releasing intrauterine system (LNG-IUS) in the treatment of endometrial hyperplasia–a long-term follow-up study. Eur J Obstet Gynecol Reprod Biol.

[90] Gallos ID, Krishan P, Shehmar M, Ganesan R, Gupta JK (2013). LNG-IUS versus oral progestogen treatment for endometrial hyperplasia: a long-term comparative cohort study. Hum Reprod.

[91] Gallos ID, Krishan P, Shehmar M, Ganesan R, Gupta JK (2013). Relapse of endometrial hyperplasia after conservative treatment: a cohort study with long-term follow-up. Hum Reprod.

[92] Cholakian D, Hacker K, Fader AN, Gehrig PA, Tanner EJ (2016). Effect of oral versus intrauterine progestins on weight in women undergoing fertility preserving therapy for complex atypical hyperplasia or endometrial cancer. Gynecol Oncol.

[93] Kim MK, Seong SJ, Kim JW, Jeon S, Choi HS, Lee IH, Lee JH, Ju W, Song ES, Park H (2016). Management of endometrial hyperplasia with a levonorgestrel-releasing intrauterine system: a Korean Gynecologic-Oncology Group Study. International journal of gynecological cancer : official journal of the International Gynecological Cancer Society.

[94] Marnach ML, Butler KA, Henry MR, Hutz CE, Langstraat CL, Lohse CM, Casey PM (2017). Oral progestogens versus levonorgestrel-releasing intrauterine system for treatment of endometrial intraepithelial neoplasia<sup/>. J Womens Health (Larchmt).

[95] Haoula ZJ, Walker KF, Powell MC (2011). Levonorgestrel intra-uterine system as a treatment option for complex endometrial hyperplasia. Eur J Obstet Gynecol Reprod Biol.

[96] Kim MK, Seong SJ, Kim YS, Song T, Kim ML, Yoon BS, Jun HS, Lee YH: Combined medroxyprogesterone acetate/levonorgestrel-intrauterine system treatment in young women with early-stage endometrial cancer. *American journal of obstetrics and gynecology* 2013, 209(4):358 e351–354. 10.1016/j.ajog.2013.06.031 23791687

[97] Dolapcioglu K, Boz A, Baloglu A: The efficacy of intrauterine versus oral progestin for the treatment of endometrial hyperplasia. A prospective randomized comparative study. *Clin Exp Obstet Gynecol* 2013, 40(1):122–126. 23724525

[98] Orbo A, Arnes M, Hancke C, Vereide AB, Pettersen I, Larsen K (2008). Treatment results of endometrial hyperplasia after prospective D-score classification: a follow-up study comparing effect of LNG-IUD and oral progestins versus observation only. Gynecol Oncol.

[99] Ismail MT, Fahmy DM, Elshmaa NS (2013). Efficacy of levonorgestrel-releasing intrauterine system versus oral progestins in treatment of simple endometrial hyperplasia without atypia. Reprod Sci.

[100] Karimi-Zarchi M, Dehghani-Firoozabadi R, Tabatabaie A, Dehghani-Firoozabadi Z, Teimoori S, Chiti Z, Miratashi-Yazdi A, Dehghani A (2013). A comparison of the effect of levonorgestrel IUD with oral medroxyprogesterone acetate on abnormal uterine bleeding with simple endometrial hyperplasia and fertility preservation. Clin Exp Obstet Gynecol.

[101] El Behery MM, Saleh HS, Ibrahiem MA, Kamal EM, Kassem GA, Mohamed Mel S (2015). Levonorgestrel-releasing intrauterine device versus dydrogesterone for management of endometrial hyperplasia without atypia. Reprod Sci.

[102] Orbo A, Arnes M, Vereide AB, Straume B (2016). Relapse risk of endometrial hyperplasia after treatment with the levonorgestrel-impregnated intrauterine system or oral progestogens. BJOG.

[103] Kresowik J, Ryan GL, Van Voorhis BJ (2008). Progression of atypical endometrial hyperplasia to adenocarcinoma despite intrauterine progesterone treatment with the levonorgestrel-releasing intrauterine system. Obstet Gynecol.

